# The fatigue spectrum in a community-based long haul COVID cohort

**DOI:** 10.1007/s11325-025-03512-y

**Published:** 2026-01-31

**Authors:** Isabelle V. Carter, Anna May, Isabella C. Hsieh, Juliane Torer, David Rosenberg, Kingman P. Strohl

**Affiliations:** 1 Department of Psychiatry, University Behavioral Health Care, Rutgers University Robert Wood Johnson Medical School, New Brunswick, NJ USA; 2 Department of Medicine, University Hospitals Cleveland Medical Center, 111000 Euclid Ave, Cleveland, OH 44107 USA; 3 Medical Service, Louis Stokes Cleveland DVA Medical Center, 10701 East Boulevard, Cleveland, OH 44122 USA; 4 Case Western Reserve University School of Medicine, Cleveland, OH USA; 5 Department of Medicine, University Hospitals Ahuja Medical Center, Beachwood, OH USA

**Keywords:** Fatigue. COVID-19, Post-acute covid syndrome (PACS), Myalgeic encephalitis, Chronic fatigue syndrome, Anxiety, Brain fog

## Abstract

**Introduction:**

In a Long Haul COVID referral clinic we describe the primary presentations of fatigue according to the CDC 2015 criteria for myalgic encephalitis/chronic fatigue syndrome (ME/CFS).

**Methods:**

Between September 2021 and April 2022, 277 patients (61% women, 54 yrs: range 18–90 yrs) presented an average of 10 months after an acute COVID-19 infection (22% hospitalized). The clinical data were analyzed to conpare those with or without a primary or co-primary complaint of fatigue, subdivided as meeting ME/CFS criteria or not.

**Results:**

209 (73.5%) people (64% women) presented with fatigue. The Fatigue Severity Score was 5.33 (out 7) in those with 5.31 (SD1.54) vs. without 4.43 (SD1.65) a primary fatigue complaint (*p* > 0.001). Anxiety (58% vs. 38%, *p* < 0.02) and any psychiatric diagnosis (66% vs. 44%%, *p* < 0.01), but not depression itself, were overrepresented in those with Fatigue and ME/CFS. Those with prior managed sleep conditions did not increase risk for fatigue presentation. Of those with fatigue and an elevated FSS, 45/209 (21.9%) met criteria for ME/CFS. In those not meeting these criteria, associated ME/CFS symptoms were less consistent. Physical functioning by ECOG (1.88 (0.78) and 26% >2) did correlate with fatigue status. Depression was present (PHQ9 12.34 (5.95) with 63% >10) to a moderate or higher degree and was different with fatigue complaints. Brain fog (51.9%) was similar among the three categories, and correlated with FSS > 4, ECOG, and depression.

**Conclusions:**

The fatigue phenotype in those presenting with it as a primary complaint comprises 21% meeting ME/CFS criteria and 79% which do not. In all the Long Haul COVID presentations. brain fog had separate, distinguishing features. Post-COVID fatigue is a spectrum which will confound clinical trials.

**Supplementary Information:**

The online version contains supplementary material available at 10.1007/s11325-025-03512-y.

## Introduction

Three months or more after recovering from an acute COVID19 infection, some 10% experience moderate to severe fatigue, not correlating well with age or acute severity or prior hospitalization [1, 2]. In our region of northeast Ohio (Cuyahoga County) from March 2020 to May 2023 there were ~ 375000 acute COVID cases (https://usafacts.org), with a potential 37,000 cases with post-COVID symptoms that might persist over time. How to handle this load of post-acute COVID fatigue continues to be a medical challenge, given lack of physician experience with fatigue as a condition, and the lack of fundamental understanding of pathophysiology and fatigue presentations.

To provide an accounting of presentations and a broader representation of the predisposing and presenting factors, we took advantage of the creation of a Long Haul COVID clinic by our health system in September 2019. The charge was to provide primary care physicians with a post-acute COVID syndrome (PASC) referral center that could: (a) collect presenting complaints in a standard manner, (b) provide a basic assessment and have guidelines for referral to specialists, and (c) provide follow up. Before it opened, an interdisciplinary group created a clinical template accompanied by questionnaires capturing the phenomenology of fatigue, activity, depression, anxiety, and sleep as captured by the experience with residual symptoms in the Canadian 2010 outbreak of the Severe Acute Respiratory Syndrome (SARS) [3], and a list of “options” that extended to any presenting complaint.

At its start in 2019, the group focused on the complaint of fatigue, and used as a template the Myalgic Encephalitis/Chronic Fatigue Syndrome (ME/CFS), defined by the CDC in 2015 [4]. This classification had limitations but a proposed alternative syndrome, “systemic exertion intolerance disease”, did not provide criteria [5]. A recent repeat 2024 effort by the IOM on post-COVID presentations resulted in acknowledging that diagnostic importance of persistence of symptoms, collection of features, and diagnostic categories including that of ME/CFS were relevant, emphasizing that long COVID is not a diagnosis of exclusion [1].

This report details the presenting characteristics and associated conditions of patients referred to a community-based Long Haul COVID Clinic from September 2021 to April 2022. The predominant presentation was persistent fatigue following this viral infection, and the hypothesis was that it would be understood with the ME/CFS [4]. We report associations with demographic information and pre-disposing conditions, using this heuristic, and correlate these features with reports of activity, mood, sleep, and psychological factors, compared to those presenting without fatigue and those with a primary complaint of fatigue but not meeting ME/CFS criteria.

## Methods

### Study Design

#### Ethical approval

All procedures performed in studies involving human participants were in accordance with the ethical standards of the University Hospitals Institutional Review Board) and with the 1964 Helsinki declaration and its later amendments or comparable ethical standards. For this type of study formal consent of the subject is not required.

The cohort was all patients who had been evaluated in an adult Long-Haul Clinic from September 2021 to April 2022. Randomized numerical aliases were generated for each presenting patient. Patients presenting to the long COVID clinic who had a complete symptomatic recovery from acute SARS-CoV-2 infection less than 2 weeks from the start of symptoms and/or positive PCR test date were excluded. Most remaining referrals (98%) were from community practitioners from Cuyahoga and Geauga counties in northeastern Ohio. Initially, the COVID B variants were present replaced by the alpha variant by mid-December. While continuously detected at 100–120 case/day in mid-2021, there was a noticeable peak in late August of 2021 and a second one in November and December, 2021. Vaccines arrived in northeast Ohio in mid-March 2021.

Consecutive patients with complete data had a documented or strongly suspected SARS-CoV-2 infection from a hospital stay (23%) or outpatient presentation (77%) and were referred for stubborn new symptoms. In patients with more than one recorded COVID-19 infection, time course of symptoms was measured from first infection. In this cohort 98% had PCR confirmed infections, the remaining were those where the presentation and circumstances of the illness occurred within a cluster of confirmed illness in close relatives or friends. The electronic medical record was verified for patient age, sex, race, ethnicity, and BMI. Laboratory testing in the post-COVID period were reviewed using not only the records from the hospital system but also a regional network of shared information. The presenting symptoms were not better explained by post-COVID anemia, thyroid, electrolyte, or liver function abnormalities.

### Capture of presenting symptoms

Each patient was interviewed and examined by an experienced advanced practice practitioner (JT) who was supervised by an experienced senior physician (DR), with the work up informed by a panel of specialists. The presenting symptoms were extracted from the initial visit note and a complaint of “fatigue” as a primary or co-primary complaint was based on the patient’s own words. Cognitive symptoms were defined as any subjective reports of concentration difficulty, perceived memory loss, word finding difficulty, and inability to effectively multitask. Those patients with fatigue prompted the assessor to specifically ask the patient about “fatigue” vs. a tendency to dose or fall asleep. Orthostatic intolerance was defined as self-report of intermittent palpitations or dizziness, often provoked by a change in posture. The presence of a pre-existing psychiatric condition was defined as any prior encounter with a specific psychiatric assessment or a diagnosis of depression or generalized anxiety disorder before the COVID acute infection.

#### Intake questionnaires

The initial evaluation consisted of intake questionnaires on fatigue (Fatigue Severity Scale), depression (PHQ9), anxiety (GAD-7), physical functioning (ECOG), and cognitive impairment (MOCA). Scores were recorded from the initial visit note and verified in the uploaded files.

A determination of Myalgic Encephalitis/Chronic Fatigue Syndrome (ME/CFS) was based on the 2015 CDC definition [4] consisting of: the three core symptoms of fatigue lasting months after the acute infection- post-exertional exacerbation and malaise, and unrefreshing sleep- as well as either impaired memory/concentration and/or orthostatic intolerance. The complaint of post-exertional increase in fatigue was obtained from question 2 in the FSS scale as a score of >4.

#### Referrals

Referrals from the primary visit were extracted from the orders from the initial COVID clinic visit note after review of the chart. Referrals with known or laboratory evidence of thyroid, liver, renal, or blood disorders were rate (< 1%). Frequency of referrals were recorded to complementary medicine for management of fatigue as well as to the following specialties: in pulmonary, cardiology, neurology, and psychiatry.

### Data collection and analysis

To minimize variation, data abstraction was performed by a two-member team performing the review of charts. Hospitalization history, preexisting conditions, and treatment course were confirmed by abstraction of the electronic health record. Past medical history was extracted from the provider note and patient-reported history, including confirmation of pre-existing conditions active prior to COVID-19 infection. The review included cardiovascular or respiratory conditions, fibromyalgia, chronic fatigue syndrome, diabetes, mental health conditions, and history of sleep disturbances or diagnoses.

The data are presented using descriptive statistics. Demographics, past medical history, and functional questionnaires were examined in relation to a categorization of No Fatigue, Fatigue, or ME/CFS criteria, as mutually exclusive groups. Logistic and ordinal regression were used as appropriate for binary outcomes or ordered categorical outcomes, respectively. Adjustors included age, race, sex, race, body mass index (BMI), and whether patient had COVID-19 hospitalization.

Participant characteristics were summarized as mean ± standard deviation (SD), median (interquartile range, IQR), or n (%) and compared using Fisher’s exact tests for categorical variables, t-tests for normally distributed continuous variables, and Wilcoxon rank sum for continuous variables with skewed distributions.

Cross-wise associations were examined for in the intersection of the presenting complaints of fatigue, FSS scores, and ME/CFS. A separate analysis was performed for the complaint of brain fog, which was not uniquely associated with presenting fatigue, FSS, or ME/CFS.

### Results

**Fig. 1:**
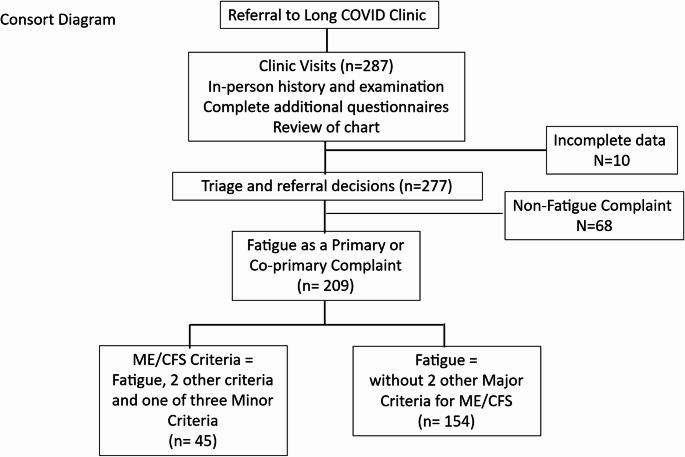
Cohort development

Shown in Fig. 1 is an accounting of the cohort in this analysis. In 10 of the initial 287 consecutive patients, questionnaire data were missing, leaving 277 subjects with complete data sets. Of these 209 (~ 75%) had fatigue as a primary or co-primary complaint.

Individuals were classified as having “ME/CFS”, “Fatigue” in those not meeting the ME/CFS criteria, and “No Fatigue” no primary presentation as “No Fatigue” (Table 1.). ME/CFS met all three Major symptoms (fatigue lasting 3–4 months or more, post-exertional malaise/fatigue, and sleep disturbances) and at least one of the following minor criteria- impaired memory/concentration or orthostatic intolerance. The “No Fatigue” group could meet some criteria other than fatigue but rates were lowest. Those meeting the criteria of ME/CSF was present in 16.5% of all and ~ 21% of those with fatigue; those with fatigue but not meeting all criteria (*n* = 164) comprised 55.6% of all referrals and 76.4% of those with the primary or co-primary complaint of fatigue.

**Table 1.** Symptoms defining ME/CFS in the cohort and in the Fatigue Categories

	Overall	ME/CFS	Fatigue	No Fatigue	*p*-value
Fatigue	209 (75.5)	45 (100.0)	164 (100.0)	0 (0.0)	**< 0.001**
Post-exertional malaise	118 (46.1)	45 (100.0)	55 (35.7)	18 (31.6)	**< 0.001**
Sleep disturbances	130 (46.9)	45 (100.0)	63 (38.4)	22 (32.4)	**< 0.001**
Impaired memory/concentration	178 (64.3)	36 (80.0)	113 (68.9)	29 (42.6)	**< 0.001**
Orthostatic intolerance	101 (36.5)	32 (71.1)	55 (33.5)	14 (20.6)	**< 0.001**

**Fig. 2:**
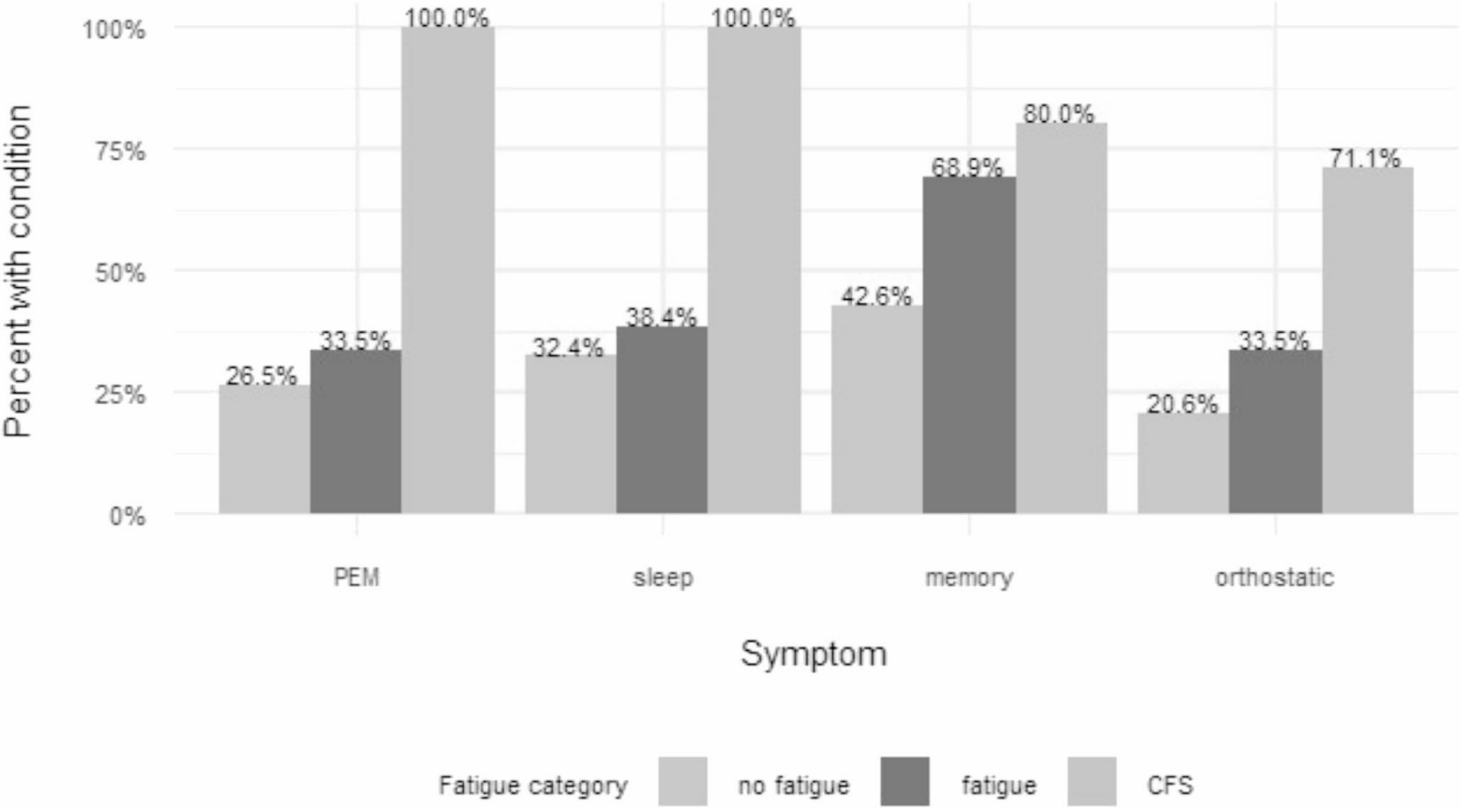
Symptoms by fatigue classification were different among the categories. Fatigue was present in present in those without this as a major complaint and, as expected, was highest in those with all defining traits of ME/CFS. Similar trends were present for general complaints of sleep and orthostatic complaints. Memory disturbances was the only trait that showed a stepwise change among the three categories

Fatigue was present in present in those without this as a major complaint and, as expected, was highest in those with all defining traits of ME/CFS. (Fig. 2). Similar trends were present for general complaints of sleep and orthostatic complaints. In the “Fatigue” group, minor criteria rates were intermediate between ME/CFS and No Fatigue. Memory disturbances was the only trait that showed a stepwise change among the three categories. Compared to the symptom of loss-of-focus which was similar among categories, poor memory was noted least often in ME/CFS. While dyspnea was common in the cohort, it was not related to the major new post-infection symptoms at presentation in this cohort.

Patients could report more than one complaint. Other symptoms at presentation are presented in Supplemental Fig. 1S, according to the ME/CFS status. While dyspnea was common in the cohort, it was not related to fatigue status. Of the new symptoms represented by more than 20% of the cohort, those of dyspnea, myalgia, new psychiatric issues, and GI distress were different among those with and without fatigue as a primary or co-primary complaint. Brain fog, often mentioned in post-COVID was greatest in the Fatigue rather than ME/SFS category, and ts correlates will be discussed below. Headache that had worsened was present in 36% and overrepresented in those with ME/CFS and Fatigue; new migraine was uncommon (3%) but not present in those with No Fatigue (*p* = 0.032).

Demographics this table (Table 2.) illustrates a population that is late middle age, more female, but otherwise is reflective of Northeast Ohio in age and BMI. There was no significant differences in these demographics among the three categories.

**Table 2.** Demographics of the cohort and in the fatigue categories

	Overall	ME/CFS	Fatigue	No Fatigue	*p*-value
n	277	45	164	68	
Age (years)	51.25 ± 14.01	49.09 ± 12.25	51.90 ± 13.37	51.10 ± 16.48	0.49
Female sex	183 (66.1)	33 (73.3)	111 (67.7)	39 (57.4)	0.17
Race					0.46
White	210 (78.4)	36 (85.7)	127 (79.4)	47 (71.2)	
Black	52 (19.4)	5 (11.9)	30 (18.8)	17 (25.8)	
Other	6 (2.2)	1 (2.4)	3 (1.9)	2 (3.0)	
Latino	6 (2.3)	0 (0.0)	5 (3.2)	1 (1.5)	0.42
BMI (kg/m^2^)	29.80 [26.38, 37.26]	29.29 [26.06, 34.00]	30.67 [26.65, 38.82]	29.52 [26.33, 33.25]	0.25

If one accepts a normal scores of < 4, the average of the 9 scores for FSS was high, with 67% having values > 4.5 and 29% values of > 6, suggesting moderate to severe levels of fatigue, and were incrementally elevated with addition criteria of ME/CFS (Table 3.). Overall, 32% MOCA scores had mild cognitive impairment or worse- values 25 or less, but were not significant if analyzed by fatigue categorization. Depression scales were elevated (PHQ9) at 11.5 with 30% >14, a moderate or higher range; and showed at similar pattern. Likewise, the anxiety scale (GAD7) was high (41% >10), and 29% of those > 12, a moderate to severe range. For this entire group, questionnaire reports of cognitive impairment physical functioning, depression, and anxiety were present in about a third of persons. MOCA and GAD7 values were no different across categories. Physical functioning (ECOG) had a third (33% >2) in a restricted range; no patient was bed-bound, yet there was a difference among categories with fatigue presentation, but the change correlated with the incremental addition of ME/CFS criteria.

**Table 3.** Questionnaire result for the cohort and the fatigue categories

	Overall		ME/CFS				Fatigue			No fatigue	*p*-value
FSS	5.33 [4.00, 6.40]				6.50 [5.39, 6.89]		5.44 [4.26, 6.33]			3.39 [2.22, 5.28]	**< 0.001**
FSS > 4	192 (73.0)				42 (95.5)		123 (79.4)			27 (42.2)	**< 0.001**
MOCA	26.00 [24.00, 28.00]		26.00 [24.25, 28.00]				26.00 [25.00, 28.00]			27.00 [24.00, 28.00]	0.93
PHQ9	11.47 ± 6.41		14.43 ± 6.44				11.86 ± 5.95			8.39 ± 6.32	**< 0.001**
GAD7	8.00 [4.00, 13.00]		10.00 [3.75, 16.25]				8.00 [5.00, 13.00]			5.00 [2.00, 13.00]	0.06
ECOG	1.58 [1.25, 2.08]		1.92 [1.56, 2.69]				1.60 [1.25, 2.15]			1.29 [1.00, 1.58]	**< 0.001**

#### Prior conditions

**Fig. 3:**
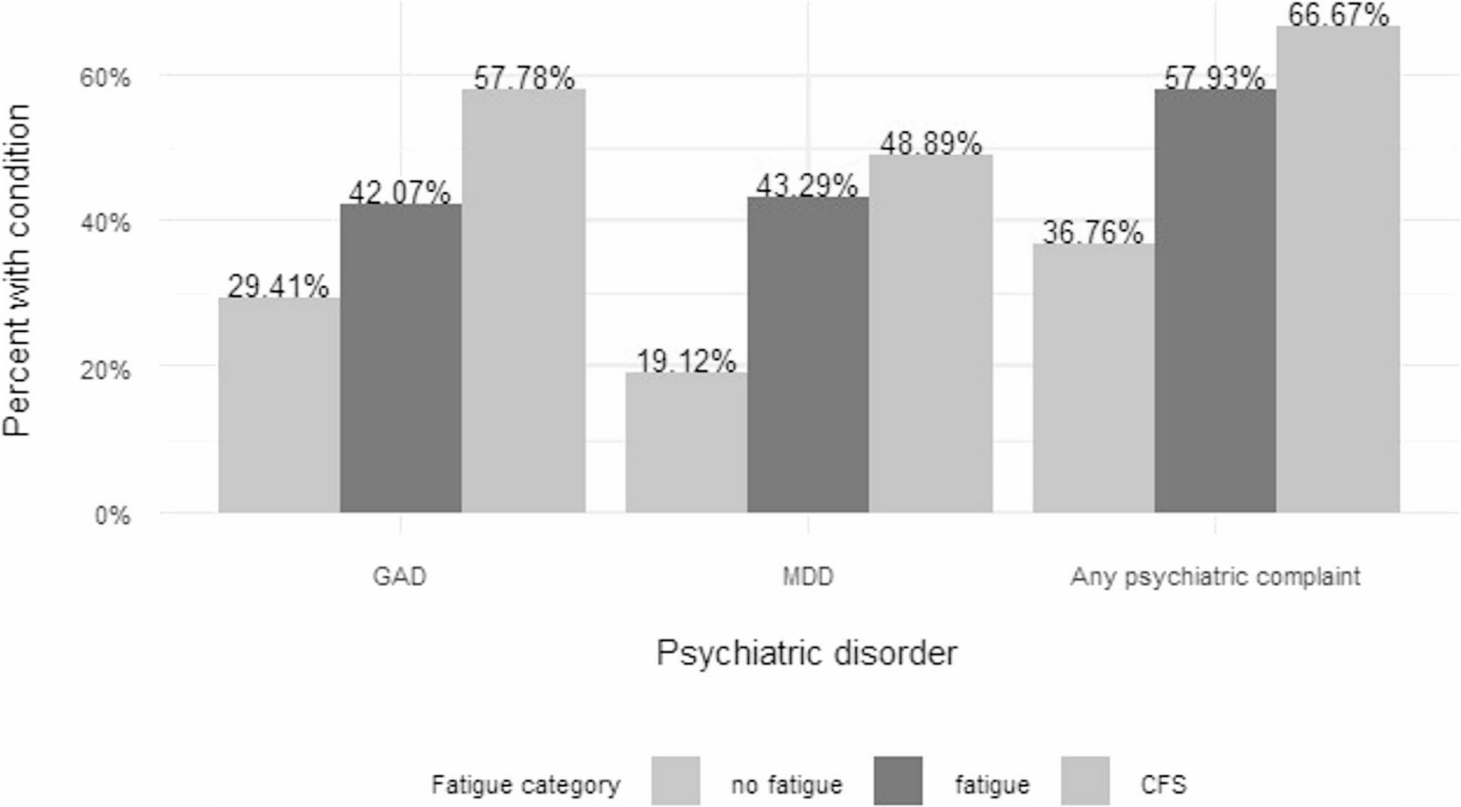
History of psychiatric comorbidity according to fatigue category. Significant predisposing conditions for ME/CFS were preexisiting psychiatric history 0.001 (n=133). history of Generalized Anxiety Disorder(GAD)0.011 (n=115), Major Depressive Disorder (MDD) history 0.001 (n=106), and any psychiatric complaint in the past

Many patients had a potentially relevant prior condition (Table 4.). Those significant were management of a psychiatric condition present in 48% of referrals with sub-groups of generalized anxiety and major depressive disorder. Insomnia (8.3%) and restless leg syndrome (3.6%) did not track by fatigue presentation. A history of a sleep problem or disorder present before the acute infection was not significantly different (0.086). A specific history of sleep disordered breathing (*n* = 63 or 23% of the sample) A prior condition of sleep apnea or sleep disordered breathing was associated with fatigue (beta 0.68, 95%CI:0.13,1.24), however the association was no longer significant after adjusting for covariates (beta 0.53, 95%CI: −0.06,1.13). A history of diabetes Type 2 (*n* = 37, 13%) was different, over-represented in the Fatigue categories vs. No Fatigue (0.014). Less common conditions < 10% were not different among groups, and included arthritis (7.2%), cancer 7%), heart failure (7%), neuropathy (96%), liver disease 4%), and atrial fibrillation (3%). A history of fatigue management was present in 3%, but differences among fatigue as a prior condition did not reach significance. A prior history of MDD and GAD were present in all No Fatigue and Fatigue categories in those with a history of MDD or Anxiety disorder, and greater the presence of either co-morbidity Fig. 3.

**Table 4.** Prior conditions for the cohort and for the fatigue categories

	Overall	ME/CFS	Fatigue	No fatigue	*p*-value
Hypothyroid	46 (16.6)	11 (24.4)	26 (15.9)	9 (13.2)	0.27
Neuropathy	16 (5.8)	3 (6.7)	12 (7.3)	1 (1.5)	0.21
Autoimmune disease	39 (14.1)	9 (20.0)	25 (15.2)	5 (7.4)	0.13
Insomnia	23 (8.3)	6 (13.3)	10 (6.1)	7 (10.3)	0.24
SDB	63 (22.7)	7 (15.6)	47 (28.7)	9 (13.2)	**0.02**
MDD	106 (38.3)	22 (48.9)	71 (43.3)	13 (19.1)	**0.001**
GAD	115 (41.5)	26 (57.8)	69 (42.1)	20 (29.4)	**0.01**
Any psychiatric history	133 (48.0)	30 (66.7)	84 (51.2)	19 (27.9)	**< 0.001**
Fibromyalgia	32 (11.6)	4 (8.9)	23 (14.0)	5 (7.4)	0.29
Diabetes	37 (13.4)	8 (17.8)	27 (16.5)	2 (2.9)	**0.01**
Heart failure	18 (6.5)	0 (0.0)	14 (8.5)	4 (5.9)	0.12
Atrial fibrillation	8 (2.9)	1 (2.2)	5 (3.0)	2 (2.9)	0.96
Asthma	58 (20.9)	7 (15.6)	40 (24.4)	11 (16.2)	0.24
Arthritis	20 (7.2)	3 (6.7)	14 (8.5)	3 (4.4)	0.54
Liver disease	12 (4.3)	0 (0.0)	11 (6.7)	1 (1.5)	0.06

#### Brain fog

**Fig. 4:**
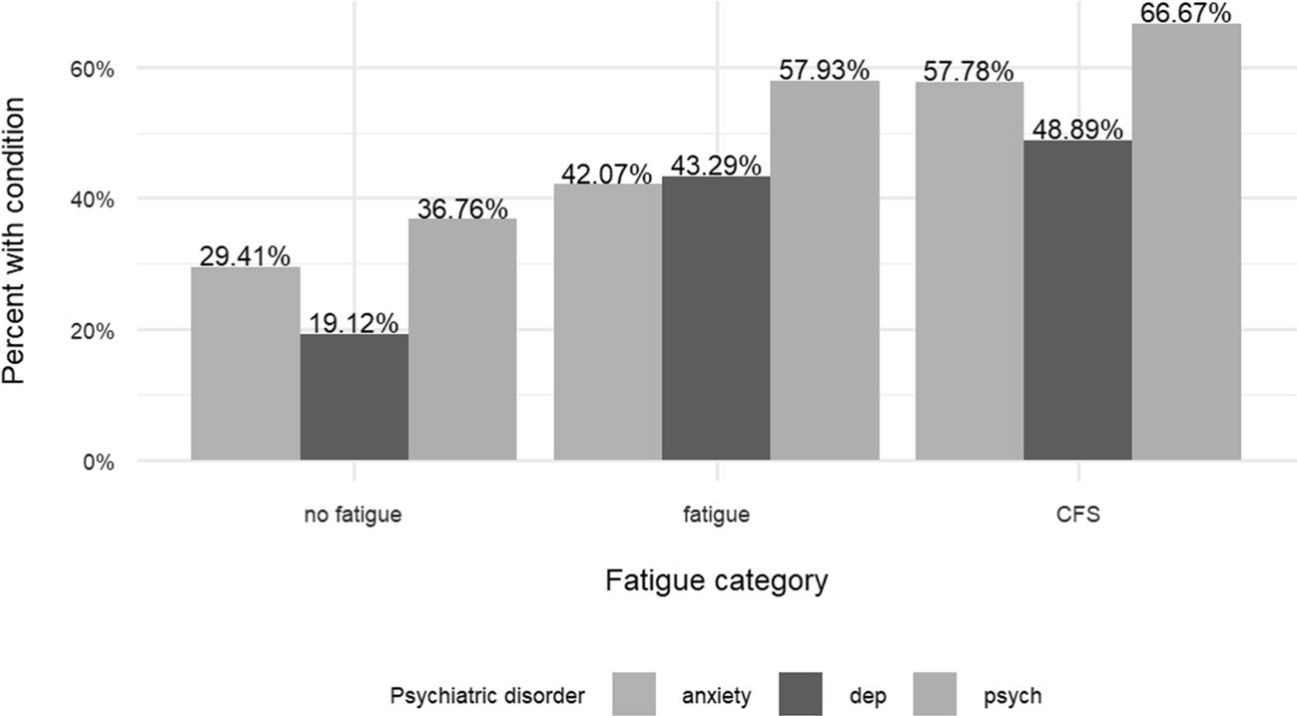
For the complaint of Brain Fog, this figure indicates the other symptoms as a percent of the cohort, according to the ME/CFS status. There was no differential presentation of the complaint of brain fog

**Fig. 5:**
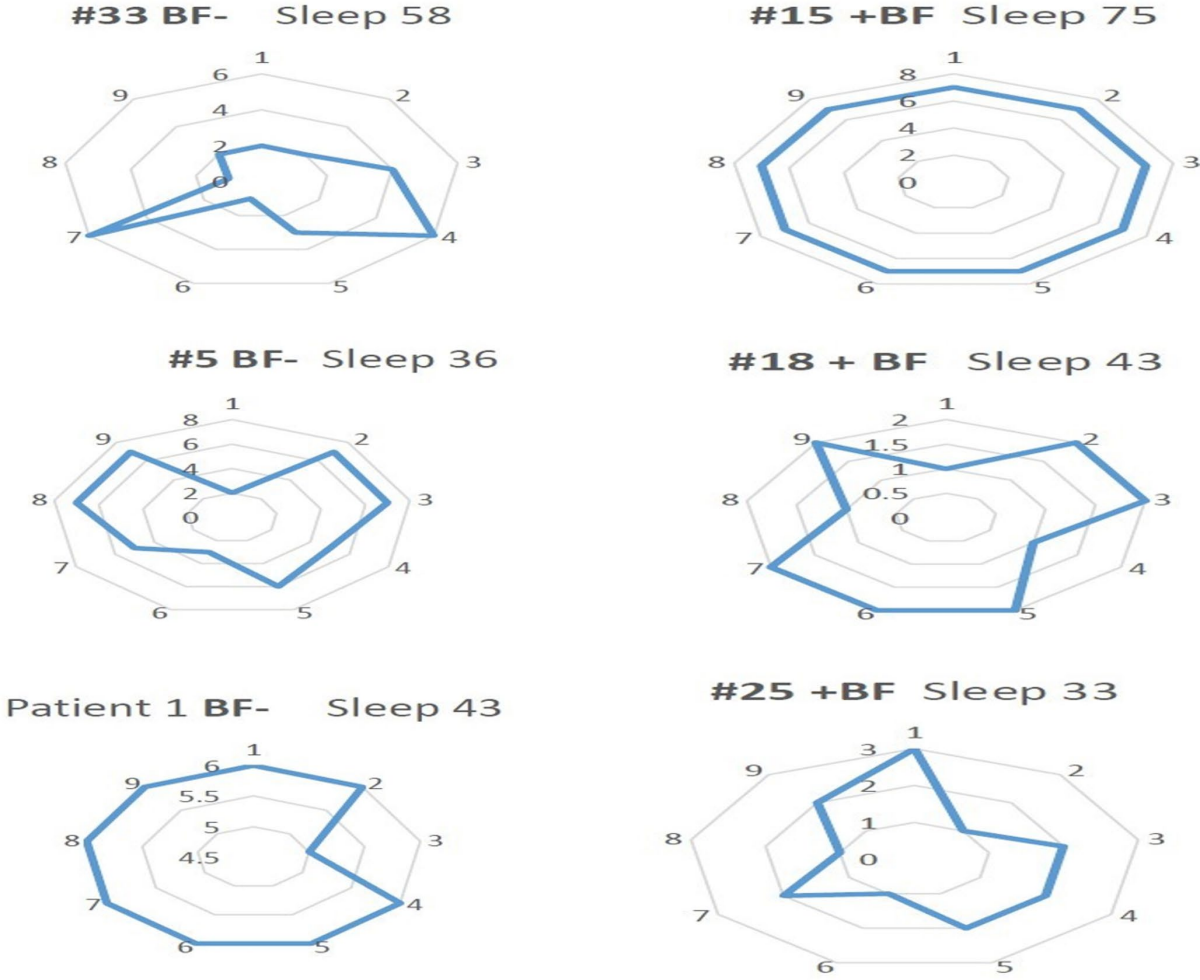
Shown are radar plots in 6 individuals with the pokes representing the values in the FSS. Each plot is for an individual patient (#), with the values for Sleep satisfaction (expressed as % maximum). Brain fog is represented as negative (BF-) or postive (+BF) report

A complaint of brain fog, often mentioned as a common symptom in post-COVID fatigue, was present in 27% of those without fatigue, and was relatively common, 51.9% (*n* = 144) of patients and was not associated with any demographic feature (Fig. 4) Supplemental Table 1S shows its association with Fatigue categories. The FSS and PHQ were higher in those reporting Brain Fog. It was however somewhat more reported in those with headache (29% vs. 43%, *p* = 0.017) and with rash (*n* = 21, 12% vs. 2%, *p* = 0.003). Brain Fog did not correlate with subcategories of the FSS was inconsistent (Fig. 5), as it could be seen in severe or mild fatigue without an association with any of the three fatigue categories.

Intake questionnaires also included pre-test probability questionnaires and follow-up on symptoms of heavy snoring, choking during sleep, waketime sleepiness, insomnia, and a BMI > 30. Eighty-five patients met a prespecified threshold for ordering a home sleep test and completed the study- 23 wit ME/CFS, 50 with fatigue, and 12 without a primary complaint of fatigue. In all categories there was no significant difference in measures of Ahi at 3% or 4% criteria or slepe time spent < 89%. AHI average was ~ 6per hour, but individuals with and AHI of 5–15,15–30, or > 30 were equally present in all three categories (*p* = 0.75).

## Discussion

While all notable post-COVID symptoms were present in this cohort, new onset fatigue of moderate severity was most often present in this ambulatory, referral population, assessed ~ 10 months after an acute presentation. There was no defining demographic for presentation. A fifth met 2015 criteria for ME/CFS and was accompanied by a higher level of self-reported fatigue as well as other defining traits. A prior history of anxiety and psychiatric management, but not depression, was enriched in those with post-COVID fatigue compared to those without fatigue. In contrast, a prior diagnosis of sleep disorders, specifically sleep apnea and insomnia, contributed little to the presentation of post-COVID fatigue report. The summary is that the fatigue presentation is a spectrum rather than a specific presentation, and that at one end of the spectrum is the condition of ME/CFS; however, other presentations of fatigue have overlapping minor symptoms. Brain fog elicited as a binary trait is distributed widely in the cohort and non-specific to fatigue severity or category per se. Such distinctions need to be understood when treatments are tested as the origins of the traits are yet clear.

While nearly 75% of the referrals were for “other fatigue” (R53.83), 21.9% met the more strict 2015 CDC ME/CFS criteria. Many others met ME/CFS criteria (G93.32). There is now a clinical code for post-COVID condition (U09.9) that has a broad tolerance in application for fatigue in patients following this viral infection. This results have not been helpful in clarifying definitions but have encouraged a more global tolerance and a more positive label for diagnostic reasoning, preferable to the codes for Functional Neurologic Disorder (2025 ICD-DM F44.6) or Conversion Disorder (F44.4). None of these diagnoses were used for referral or management in this cohort.

The post-COVID cohort here was consistent with current views that the chronic brain dysfunction occurs by “inflammation” caused by the acute illlness [6] and a persistence is documented plasma samples in long COVID [7]. Besides the convenience sample approach, control studies and normal values are not collected with attention to baseline symptoms. We detect signals for cognitive issues in those who had no fatigue. The referral nature of this collection permits a snapshot of the post-COVID conditions. In this group, MOCA scores suggested 32% had mild cognitive impairment or worse. Physical functioning by ECOG (1.88 (0.78) and 26% >2) was restricted, but no patient was bed-bound. Depression scales were elevated (PHQ9 12.34 (5.95) with 63% >10) in a moderate or higher degree. Anxiety scale (GAD7) was high (>10) in 29%, a moderate to severe degree. These scores are present in older studies of ME/CFS and may not be unreasonable for those whose social and personal life is affected by fatigue [8]. While these scales and others like PROMIS constructs are not specific for COVID, and can be seen in other disorders which affect sleep, muscular systems, autonomic tone, etc., the model of long COVID is described as a distinct life inflection point. Brain structural damage is present [9], but linking these reports and somatic complaints/markers to brain connectivity [10, 11] should be a basic approach to understanding the current illness and interventions [12].

The findings from this geographic region of the United States generally reflect the literature on post-COVID syndromic presentations. One of the first Midwest samples emphasized found a third of patients had returned to unrestricted work and those who could not had relatively normal laboratory and imaging tests despite debilitating symptoms in fatigue, perceived cognitive impairment, and mood [13]. The emphasis on fatigue is attributed to the objective of the clinic to provide a referral site for primary care practitioners for this complaint rather than select specialty-specific referrals which asked for referrals only for those with pulmonary, cardiac, neurologic, and gastroenterological complaints.

Brain Fog (F44.8) was enriched in the ME/CFS group but not enough to have a specific impact as it was present in patients with or without fatigue to a similar extent. Prior publications have suggested that its presence in long COVID increases all personal and social domains of the fatigue condition [14]. Another report tried to separate out ME/CFS and post-COVID presentation and concluded that while the symptomatology and cognitive patterns were similar in both groups, there was greater impairment in ME/CFS [15]. While not specific to post-COVID fatigue, a presence of “brain fog” does portend a decline in labor productivity in rehabilitation of these patients [16]. and the associated social stigma and emotional and mental health [14]. As seen in this study a high proportion seeking treated was of working age and moderate to greater functional limitation, and high levels of fatigue depression and cognitive stress [17]. Brain fog is found in many sleep disorders, some like narcolepsy which are characterized by sleepiness and some like insomnia which are accompanied by hyperarousal. A recent publication addressed the development and initial psychometric properties of a questionnaire called Fatigue and Altered Cognition Scale (the FACs) as it might apply to self-reported “central” fatigue and brain fog, and tested in in traumatic brain injury rehabilitation [18]. The items, initially identified by both patients and clinician, were better captured by a two-factor than one-factor model in comparing those with and without TBI.

In one report both physical activity and a sense of sleep health are individually associated with depressive symptoms, with sleep health mediating 19% of an association between physical activity and depression symptoms [19]. Among the three groups, poor sleep health was present, but perhaps referral bias attenutated an association of sleep health with fatigue, activity or pre-morbid conditions because the patients were ill. Sleep disorders captured by questionnaire and follow-up questions were also non-specific. Although this resulted in many (%) referrals for testing. Those with an insomnia diagnosis before the acute illness had sleep satisfaction deteriorate with prior prescribed or over-the-counter drugs no longer providing being helpful. This has been noted before in ME/CFS conditions [3]. One individual had a pre-existing diagnosis of narcolepsy and symptoms of poor sleep, insomnia, and sleepiness had become more difficult to manage with alerting medication; another was suspected but symptoms preceded the COVID acute illness. Acute COVID infection does not appear as a causal feature in the ME/CFS or fatigue group, more likely a result of the impact of sleep on rehabilitation from acute conditions. RECOVER Sleep, an NIH sponsored study, is examining the effects of sleep rehabilitation in moderate to severe long COVID.

The strength of this report is the value provided by ME/CFS criteria with its multidimensional medical and psychological assessment. It is a foundation which we hope to expand to longitudinal assessments and health outcomes in the fatigue spectrum (state vs. trait), to identify the stability of the symptoms useful to an interventional cohort. The limitations are that this is a single-site and small sample, despite the presentation commonalities with the larger data sets [20]. While vaccines were available, there is not a good handle on a a patient’s status at our time of assessment some months after the acute illness; fully vaccinated before the first COVID infection results in a lesser severity of post-COVID-19 fatigue, ageusia/hypogeusia, dizziness, tinnitus, and insomnia [21], and evidence suggests that vaccination attenuates brain inflammation and biomarkers [22]; however, prevalence of post-COVID vaccine neurocognitive symptoms are still at 15–25%. The database EMR collections on the topic, for example PHOSP-COVID [23], are based on a given reported aggregate diagnosis by an encounter, the basis for which is often hidden and the criteria collected from coding records or a combination of coding and natural language. We relied on a curated, standardized dataset to distinguish ME/CFS from Fatigue, yet the process may have erred in either direction in a specific domain to mislabel a few individuals. This effect did not prevent the correlation of stepwise associations with presenting mood and activity profiles and co-morbidity, with lowest levels of neurocognitive symptoms still detected in those who did not present with a primary or co-primary report of fatigue. Given the paucity of therapy proposed for the condition, collecting such longitudinal information for therapeutic trials may help detect endotypes of the post-COVID condition responding to therapy or resolving over time.

## Conclusions

This boutique collection adds to the literature by providing a focus for defining post-COVD fatigue within the prior framework of ME/CFS and showing that this presenting complaint is a spectrum. In addition, a report of brain fog does not exclusively map to fatigue. There are predisposing factors of anxiety and pre-existing psychiatric conditions but again these are non-specific. While many were offered additional tests, like home sleep studies, and treatment approaches, i.e. wellness programs, the use and outcomes are not defined here. Finally, this effort serves to focus thinking about varying fatigue presentations and thoughtfully engage both clinicians and pre-clinical investigators in the process of developing interventional protocols for this complaint.

## Supplementary Information

Below is the link to the electronic supplementary material.

Supplementary Material 1 (DOCX 101 KB)

Supplementary Material 2 (DOCX 19.4 KB)

## Data Availability

Deidentified data abstraction sheets are available upon a reasonable request to the corresponding author. There are no data archived in supplemental form.
