# Supplementary figures and images for: The fatigue spectrum in a community-based long haul COVID cohort
Source: Sleep Breath. 2026 Jan 31;30(1):27. doi: 10.1007/s11325-025-03512-y (PMC12860841; doi:10.1007/s11325-025-03512-y)

Figure 1S. Shown are the other symptoms as a percent of the cohort, according to the ME/CFS status.


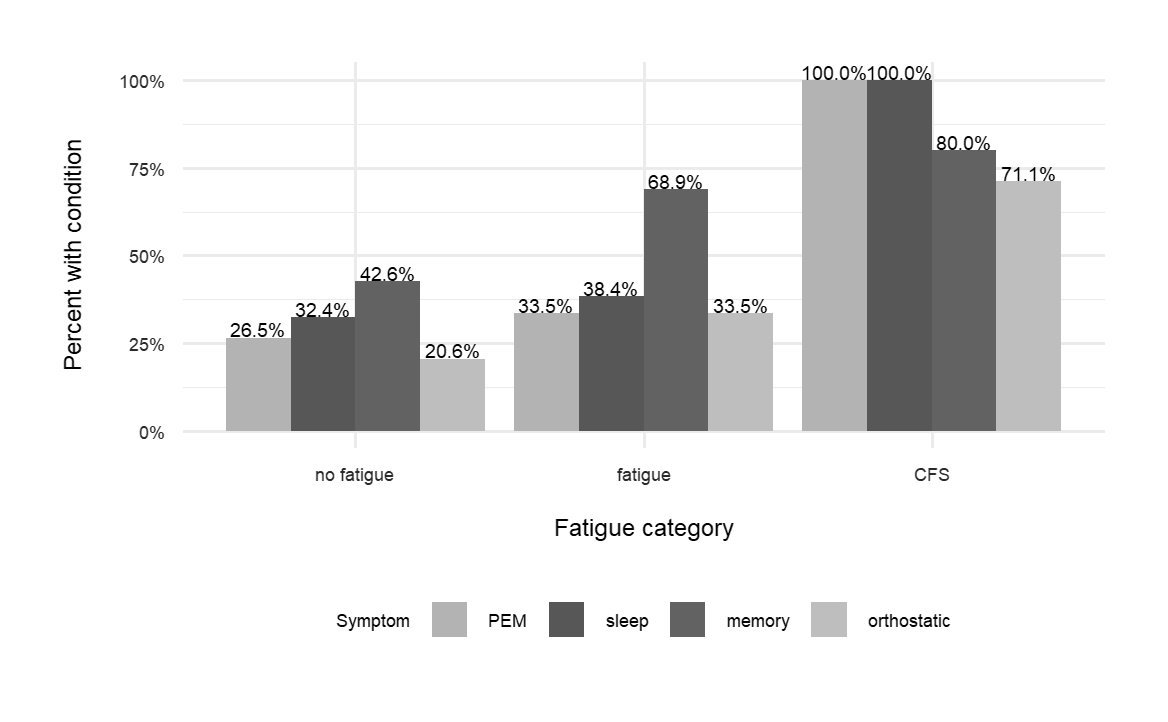

Supplement: Supplementary file 1 — Supplementary Material 1 (DOCX 101 KB) [file 11325_2025_3512_MOESM1_ESM.docx]
