# Supplementary material for: The fatigue spectrum in a community-based long haul COVID cohort
Source: Sleep Breath. 2026 Jan 31;30(1):27. doi: 10.1007/s11325-025-03512-y (PMC12860841; doi:10.1007/s11325-025-03512-y)
Supplement: Supplementary file 2 — Supplementary Material 2 (DOCX 19.4 KB) [file 11325_2025_3512_MOESM2_ESM.docx]

Table 1S. Associations among Collected Data and the Absence or Presence of Brain Fog

|  |  |  | Overall | No brain fog | Brain fog | p |
| --- | --- | --- | --- | --- | --- | --- |
|  |  | n | 277 | 133 | 144 |  |
|  |  | age (mean (SD)) | 51.25 (14.01) | 52.46 (15.22) | 50.12 (12.75) | 0.167 |
|  | Demographics | female.f = 1 (%) | 183 (66.1) | 85 (63.9) | 98 (68.1) | 0.548 |
|  |  | race.f (%) |  |  |  | 0.685 |
|  |  | white | 210 (78.4) | 99 (76.2) | 111 (80.4) |  |
|  |  | black | 52 (19.4) | 28 (21.5) | 24 (17.4) |  |
|  |  | other | 6 (2.2) | 3 (2.3) | 3 (2.2) |  |
|  |  | latino.f = 1 (%) | 6 (2.3) | 3 (2.3) | 3 (2.2) | >0.99 |
|  |  | bmi (median [IQR]) | 29.80 [26.38, 37.26] | 29.52 [25.71, 34.26] | 30.20 [27.02, 38.68] | 0.07 |
|  |  | covidHosp.f = 1 (%) | 71 (25.6) | 29 (21.8) | 42 (29.2) | 0.206 |
|  | Scales | fss (median [IQR]) | 5.33 [4.00, 6.40] | 4.83 [3.19, 6.30] | 5.67 [4.58, 6.50] | 0.002 |
|  |  | sleepSatisfaction.f = 1 (%) | 151 (57.4) | 76 (61.8) | 75 (53.6) | 0.223 |
|  |  | moca (median [IQR]) | 26.00 [24.00, 28.00] | 27.00 [25.00, 28.00] | 26.00 [24.00, 27.00] | 0.15 |
|  |  | sri (mean (SD)) | 24.50 (6.36) | 23.81 (5.68) | 25.11 (7.01) | 0.56 |
|  |  | sdis (mean (SD)) | 21.44 (4.41) | 21.88 (5.20) | 21.06 (3.67) | 0.596 |
|  |  | phq (mean (SD)) | 11.47 (6.41) | 10.31 (6.47) | 12.46 (6.20) | 0.007 |
|  |  | gad7 (median [IQR]) | 8.00 [4.00, 13.00] | 7.00 [3.00, 13.00] | 8.00 [4.00, 14.00] | 0.22 |
|  |  | ecog (median [IQR]) | 1.58 [1.25, 2.08] | 1.33 [1.08, 1.83] | 1.80 [1.42, 2.33] | <0.001 |
|  | Symptoms | fatigue.f = 1 (%) | 209 (75.5) | 83 (62.4) | 126 (87.5) | <0.001 |
|  |  | dyspnea.f = 1 (%) | 161 (58.1) | 75 (56.4) | 86 (59.7) | 0.66 |
|  |  | psychNew.f = 1 (%) | 138 (49.8) | 58 (43.6) | 80 (55.6) | 0.062 |
|  |  | anySleepProb.f = 1 (%) | 130 (46.9) | 55 (41.4) | 75 (52.1) | 0.095 |
|  |  | headache.f = 1 (%) | 100 (36.1) | 38 (28.6) | 62 (43.1) | 0.017 |
|  |  | myalgia.f = 1 (%) | 98 (35.4) | 41 (30.8) | 57 (39.6) | 0.162 |
|  |  | anosmia.f = 1 (%) | 76 (27.4) | 37 (27.8) | 39 (27.1) | 0.998 |
|  |  | palps.f = 1 (%) | 69 (24.9) | 26 (19.5) | 43 (29.9) | 0.065 |
|  |  | cough.f = 1 (%) | 68 (24.5) | 30 (22.6) | 38 (26.4) | 0.548 |
|  |  | insomnia.f = 1 (%) | 65 (23.5) | 28 (21.1) | 37 (25.7) | 0.442 |
|  |  | chest.f = 1 (%) | 63 (22.7) | 33 (24.8) | 30 (20.8) | 0.518 |
|  |  | dizzy.f = 1 (%) | 60 (21.7) | 22 (16.5) | 38 (26.4) | 0.066 |
|  |  | giDis.f = 1 (%) | 59 (21.3) | 26 (19.5) | 33 (22.9) | 0.591 |
|  |  | poorMem.f = 1 (%) | 40 (14.4) | 21 (15.8) | 19 (13.2) | 0.658 |
|  |  | focus.f = 1 (%) | 35 (12.6) | 18 (13.5) | 17 (11.8) | 0.801 |
|  |  | weakness.f = 1 (%) | 40 (14.4) | 15 (11.3) | 25 (17.4) | 0.205 |
|  |  | weightGain.f = 1 (%) | 28 (10.1) | 10 (7.5) | 18 (12.5) | 0.24 |
|  |  | gait.f = 1 (%) | 26 (9.4) | 12 (9.0) | 14 (9.7) | >0.99 |
|  |  | rash.f = 1 (%) | 21 (7.6) | 3 (2.3) | 18 (12.5) | 0.003 |
|  |  | hairLoss.f = 1 (%) | 20 (7.2) | 10 (7.5) | 10 (6.9) | >0.99 |
|  |  | hearing.f = 1 (%) | 17 (6.1) | 6 (4.5) | 11 (7.6) | 0.405 |
|  |  | edema.f = 1 (%) | 12 (4.3) | 4 (3.0) | 8 (5.6) | 0.456 |
|  |  | weightLoss.f = 1 (%) | 11 (4.0) | 5 (3.8) | 6 (4.2) | >0.99 |
|  |  | dysphagia.f = 1 (%) | 9 (3.2) | 7 (5.3) | 2 (1.4) | 0.139 |
|  |  | migraine.f = 1 (%) | 9 (3.2) | 5 (3.8) | 4 (2.8) | 0.904 |
|  |  |  |  |  |  |  |
